# Supplementary material for: An Experimental Validation Test of Ecological Coexistence Theory to Forecast Extinction Under Rising Temperatures
Source: Ecol Lett. 2024 Dec 31;28(1):e70047. doi: 10.1111/ele.70047 (PMC11687354; doi:10.1111/ele.70047)
Supplement: Supplementary file 1 — Data S1: [file ELE-28-0-s001.pdf]

Supplementary Information

An experimental validation test of ecological coexistence theory to forecast extinction under rising temperatures.

J. Christopher D. Terry[1*]

1: Department of Biology, University of Oxford, 11A Mansfield Road, Oxford, OX1 3SZ

christopher.terry@biology.ox.ac.uk 0000-0002-0626-9938

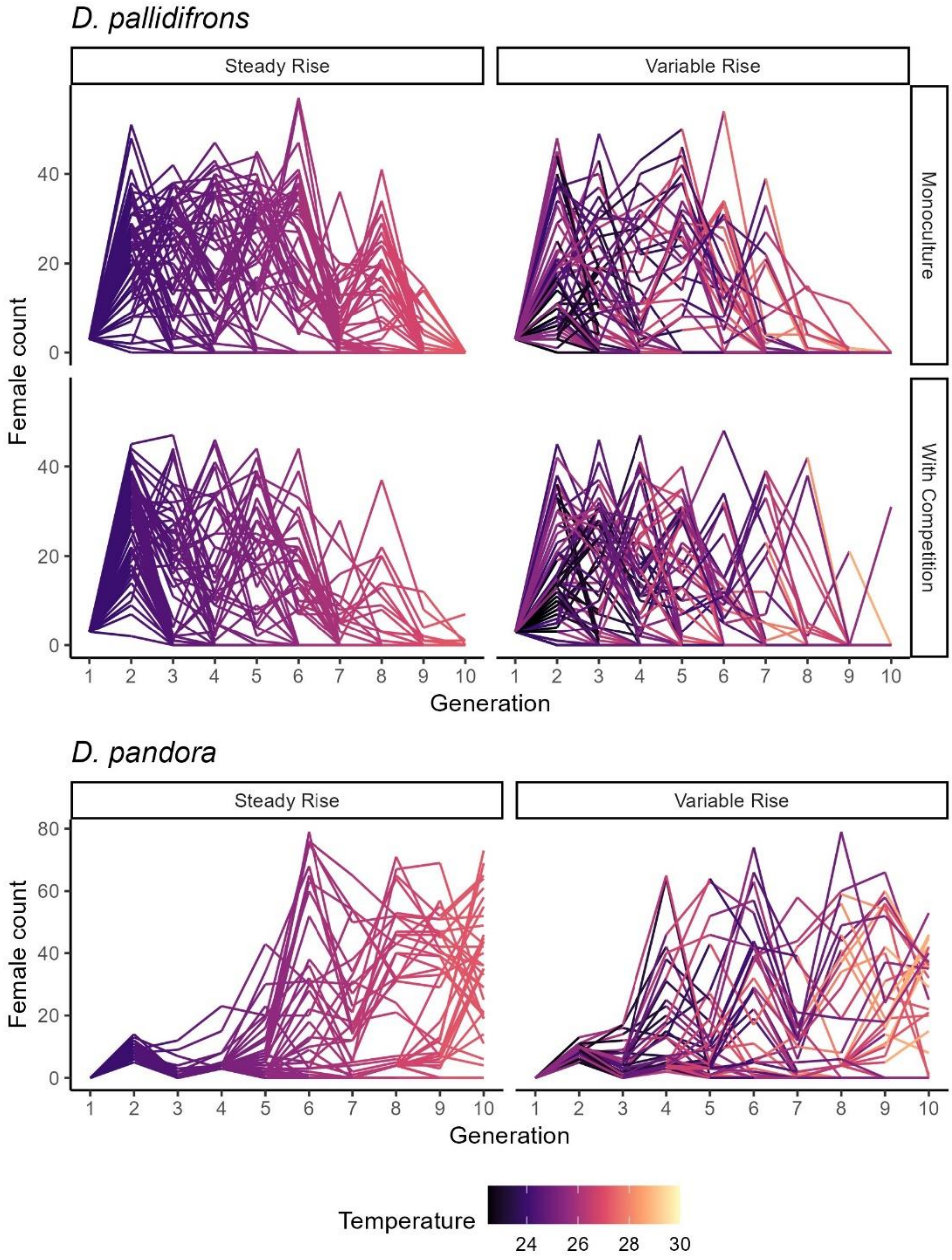

Figure S1 Raw time series of female counts for each population line during the experiment facetted by treatment and coloured by incubator temperature.

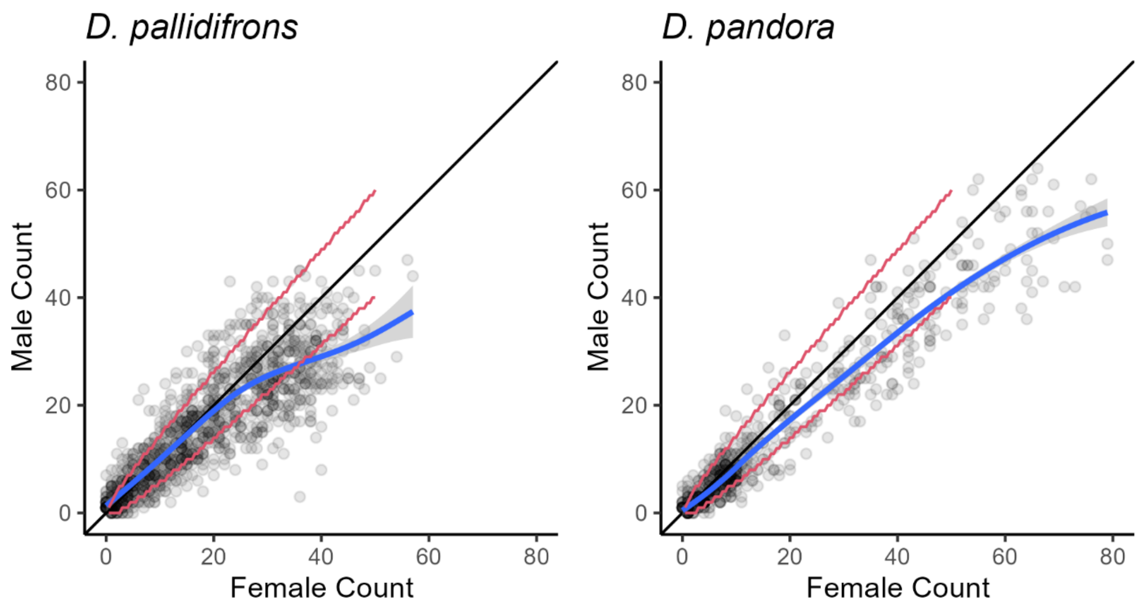

Figure S2 Sex ratio of the two species with respect to density. Red lines show 95% interval of assuming a 1:1 ratio. Blue line shows a smooth loess plot highlighting female skew at high overall densities.

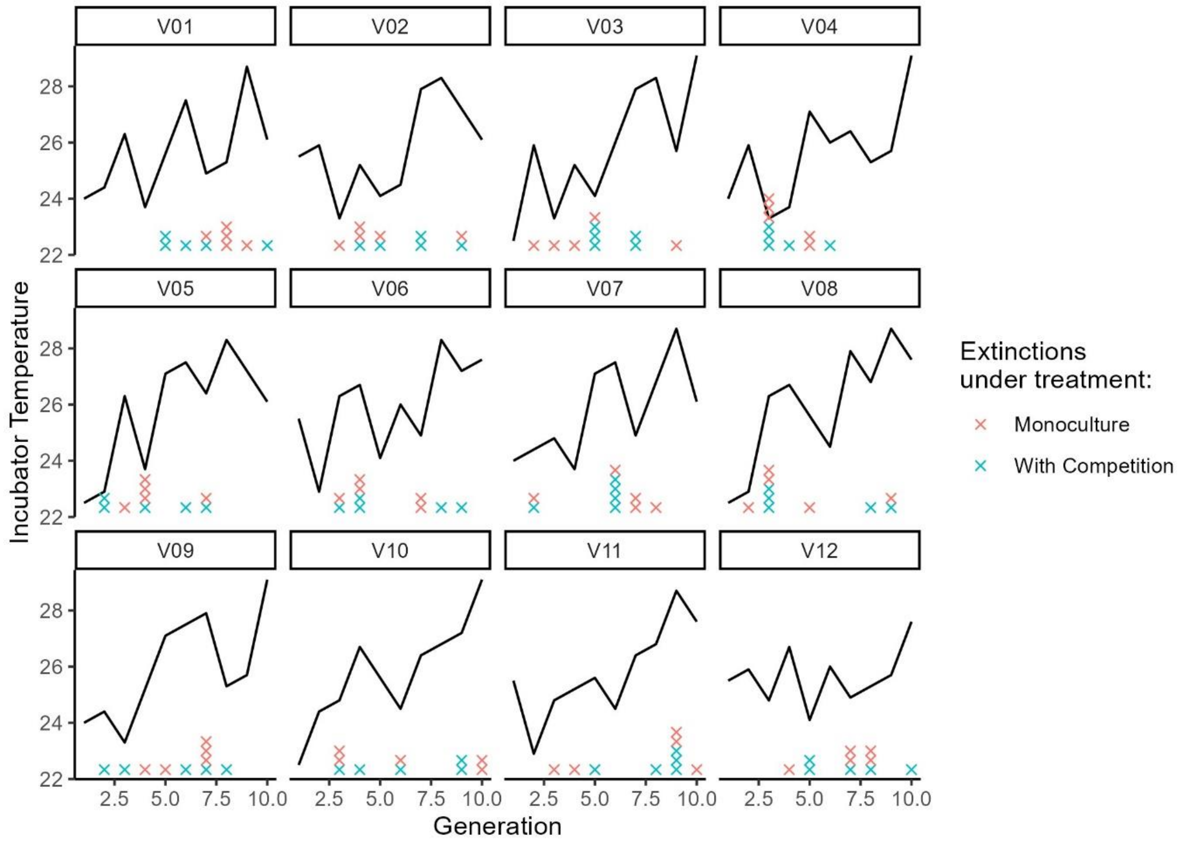

Figure S3. Extinctions within each variability trajectory within the 'Variable' temperature treatment.

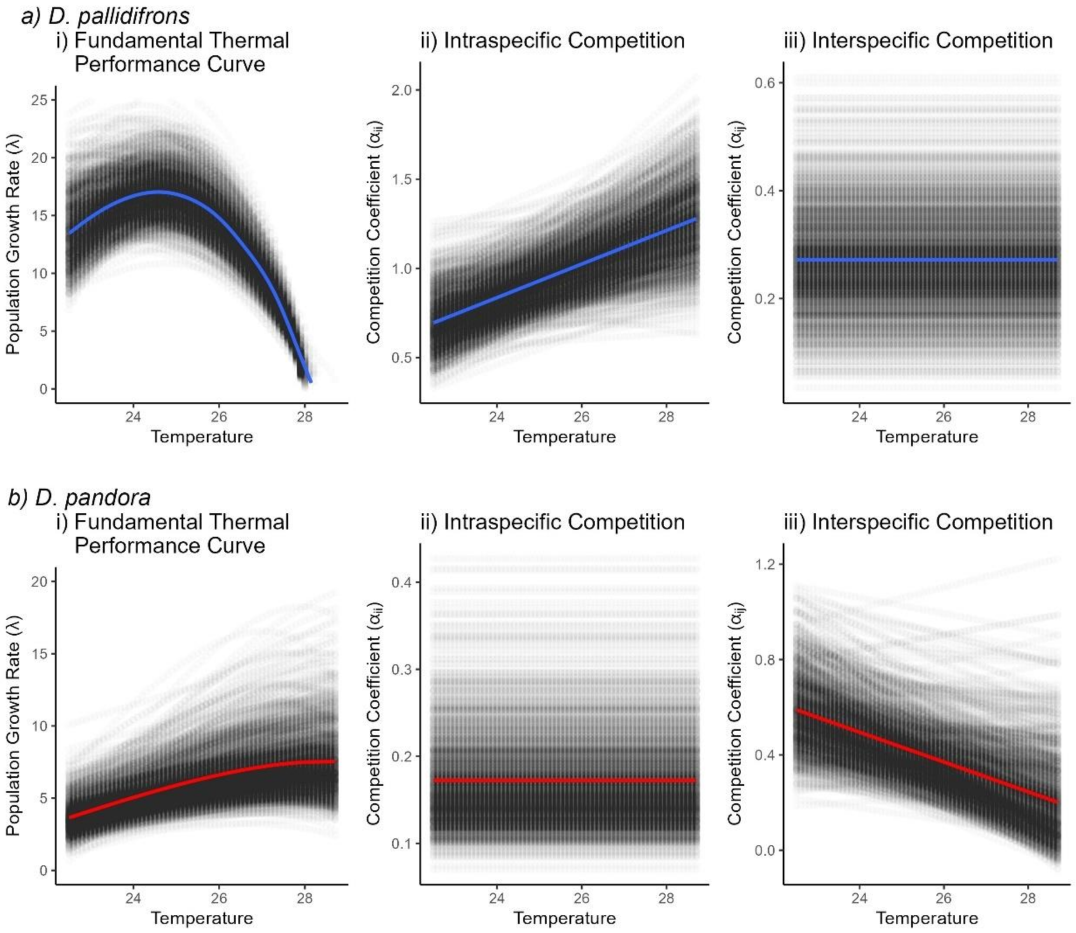

Figure S4 Posterior distribution of dependence of composite parameters (λ and α competition coefficients) on temperature for the selected model for each species. Coloured lines show a smooth fit through the posterior distribution.

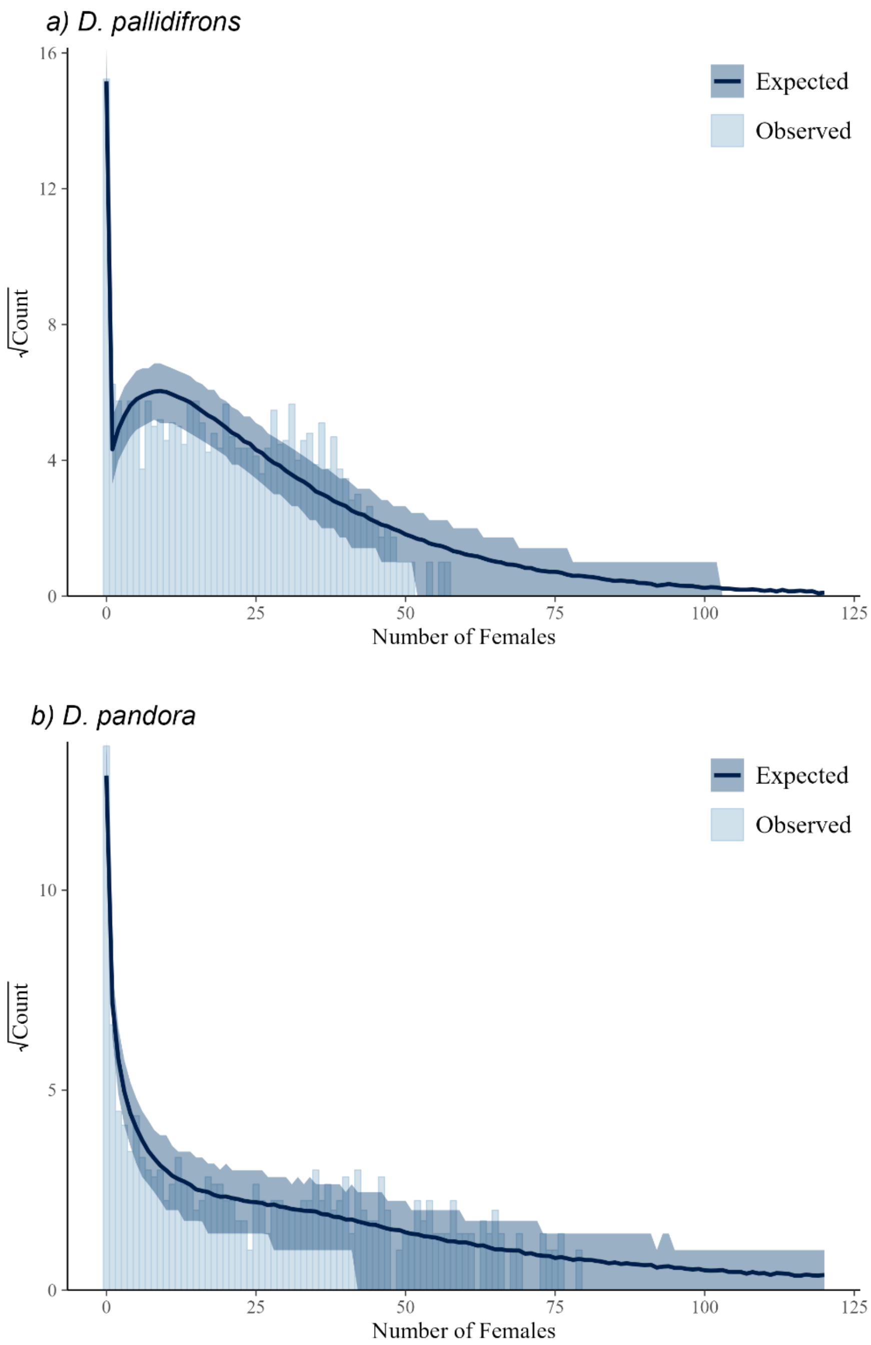

Figure S5. Posterior predictive distribution of selected model through rootograms[1]**.** Overall, the modes can perform well at low levels, including the zero-inflation, although the model predicts more high counts of both species than are observed.

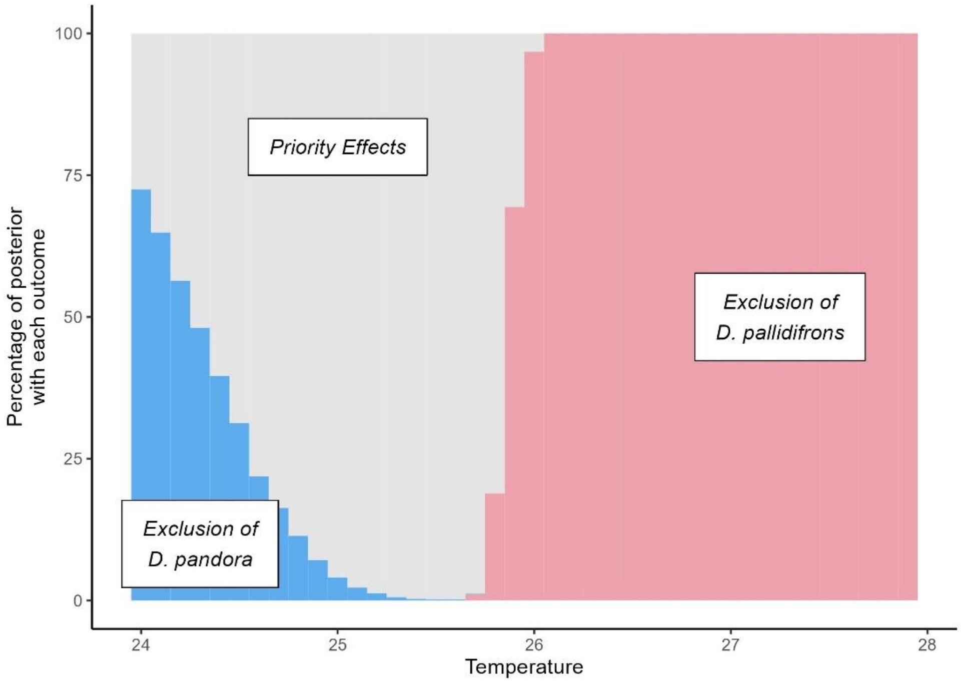

Figure S6. Coexistence predictions using the same model competition and thermal performance model structure, but a Poisson error distribution. Compared to the principal model, this alternative identifies a period of priority effects rather than coexistence and a much sharper transition. Despite poorly capturing the observed error distribution, it is notable that it gives both an accurate and precise prediction of the point of exclusion.

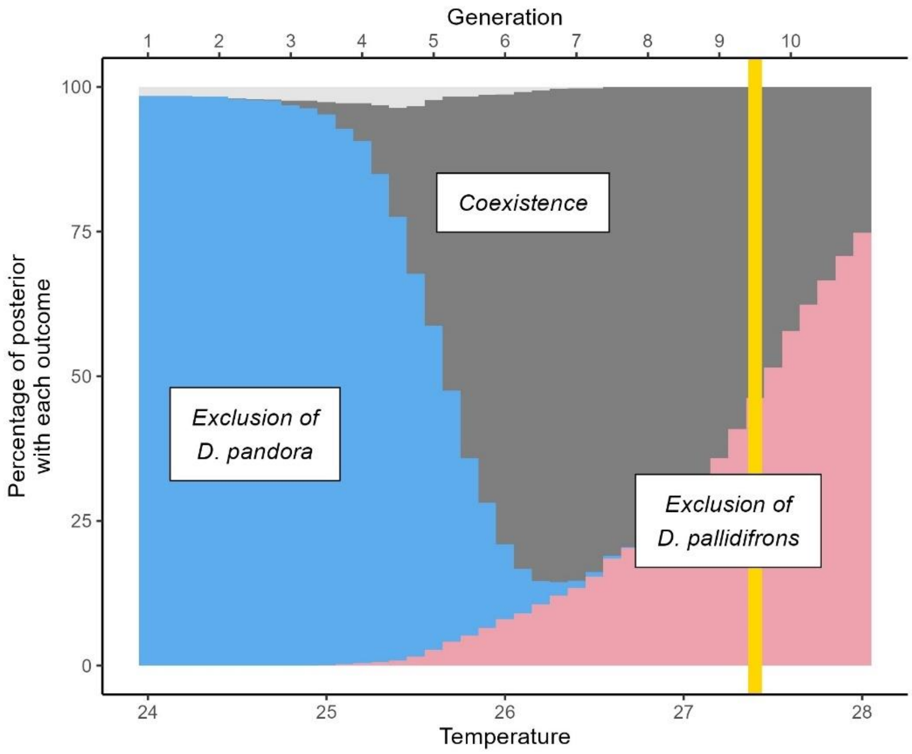

Figure S7 Coexistence predictions where *D. pallidifrons* model is trained without transition data from the 'steady rise, competition' experimental treatment used as the principal test.

Table S1. Tested competition model structures and dependencies. To prevent combinatorial explosion, models chosen to be tested were developed through sequential modification of initial candidate models based on observed fitted parameters.

Model Name (BH = Beverton-Holt)	Model formula for competition kernel	N
Lotka-Volterra (excluded due to poor convergence)	$1 + \alpha_{i,PAL}N_{PAL,t} + \alpha_{i,PAN}N_{\mathrm{PAN},t}$	**2**
BH	$\frac{1}{1 + \alpha_{i,PAL}N_{PAL,t} + \alpha_{i,PAN}N_{\mathrm{PAN},t}}$	**2**
Beta-BH	$\frac{\mathbf{1}}{\left(1 + \alpha_{i,PAL}N_{PAL,t} + \alpha_{i,PAN}N_{\mathrm{PAN},t}\right)^{\boldsymbol{\beta}}}$	**3**
Two-way Temperature dependent BH	$\frac{\mathbf{1}}{1 + (\alpha_{0i,PAL} + \alpha_{Ti,PAL}T_t)N_{PAL,t} + (\alpha_{0i,PAN} + \alpha_{Ti,PAN}T_t)N_{PAN,t}}$	**4**
Two-way Temperature dependent Beta- BH	$\frac{\mathbf{1}}{\left(1 + (\alpha_{0i,PAL} + \alpha_{Ti,PAL}T_t)N_{PAL,t} + (\alpha_{0i,PAN} + \alpha_{Ti,PAN}T_t)N_{PAN,t}\right)^{\boldsymbol{\beta}}}$	**5**
PAL-only Temperature dependent BH	$\frac{1}{1 + (\alpha_{0i,PAL} + \alpha_{Ti,PAL}T_t)N_{PAL,t} + \alpha_{i,PAN}N_{j,t}}$	**3**
PAL-only Temperature dependent Beta- BH	$\frac{\mathbf{1}}{\left(1 + (\alpha_{0i,PAL} + \alpha_{Ti,PAL}T_t)N_{PAL,t} + \alpha_{i,PAN}N_{PAN,t}\right)^{\boldsymbol{\beta}}}$	**4**
PAL-only Temperature dependent BH with log-competition	$\frac{1}{1 + (\alpha_{0i,PAL} + \alpha_{Ti,PAL}T_t)\ \log(N_{PAL,t} + 1) + \alpha_{i,PAN}\ log\ (N_{PAN,t} + 1)}$	**3**
PAL-only Temperature dependent Beta-BH with log-competition	$\frac{\mathbf{1}}{\left(1 + (\alpha_{0i,PAL} + \alpha_{Ti,PAL}T_t)\log(N_{PAL,t} + 1) + \alpha_{i,PAN}log(N_{PAN,t} + 1)\right)^{\boldsymbol{\beta}}}$	**4**

Table S2. Tested error model structures and dependencies with *brms* package R code syntax for additional clarity. This uses the negative binomial parameterisation in terms of mean (or location, μ) and a shape parameter (ϕ) such that $E[n] = \mu$ and $Var[n] = \mu + \frac{\mu^2}{\phi}$.

**Model Name (NB = Negative-Binomial)**	**Code used within *brms***
Poisson	family = poisson(link = "identity")
Negative Binomial	family = negbinomial(link = "identity")
Zero-inflated NB	family = zero_inflated_negbinomial(link = "identity")
Temperature-dependent-zero-inflated NB	family = zero_inflated_negbinomial(link = "identity")<br>additional terms: zi~PrevTemp
Zero-inflated NB with competition dependent shape	family = zero_inflated_negbinomial(link = "identity")<br>additional terms: shape~Prev_PAL_FEMALE or shape~Prev_PAN_FEMALE
Zero-inflated NB with temperature dependent shape	family = zero_inflated_negbinomial(link = "identity")<br>additional terms: shape~PrevTemp

Table S3. Relative performance of the top 25 models (out of 48 that were comparable) to fit the *D. pallidifrons* transition data. All tested models used the Briere thermal performance curve. R-hat convergence is of the most poorly converged parameter in the model.

Competition function	Error Model	ΔELPD LOO	R-hat Convergence
BHTEMP1Beta	Temperature-dependent-zero-inflated NB	0	1.001
BHTEMPBeta	Temperature-dependent-zero-inflated NB	-0.308	1.003
BHTEMP	Temperature-dependent-zero-inflated NB	-6.1	1.002
BHTEMP1 (x)	Temperature-dependent-zero-inflated NB	-6.29	1.002
BHBeta	Temperature-dependent-zero-inflated NB	-7.42	1.002
BH	Temperature-dependent-zero-inflated NB	-9.14	1.002
BHTEMPBeta	Zero-inflated NB with temperature-dependent shape	-11.7	1.014
BHTEMP1LogBeta	Temperature-dependent-zero-inflated NB	-13.4	1.002
BHTEMP	Zero-inflated NB with temperature-dependent shape	-19.4	1.002
BHTEMP1	Zero-inflated NB with temperature-dependent shape	-20.4	1.001
BHTEMP1LogBeta	Zero-inflated NB with temperature-dependent shape	-25.5	1.003
BHBeta	Zero-inflated NB with temperature-dependent shape	-25.7	1.001
BH	Zero-inflated NB with temperature-dependent shape	-28.4	1.001
BHTEMPBeta	Zero-inflated NB	-37	1.001
BHTEMP1Beta	Zero-inflated NB	-37.1	1.003
BHTEMPBeta	Zero-inflated NB with competition-dependent shape	-37.7	1.002
BHTEMP1Beta	Zero-inflated NB with competition-dependent shape	-38.3	1.003
BHTEMP	Zero-inflated NB	-42.3	1.003
BHBeta	Zero-inflated NB	-42.5	1.001
BHTEMP1	Zero-inflated NB	-42.6	1.002
BHTEMP	Zero-inflated NB with competition-dependent shape	-43.5	1.004
BHBeta	Zero-inflated NB with competition-dependent shape	-43.7	1.002
BHTEMP1	Zero-inflated NB with competition-dependent shape	-43.7	1.002
BH	Zero-inflated NB	-44.2	1.002
BH	Zero-inflated NB with competition-dependent shape	-45.5	1.002

Table S4 Relative performance of the top 25 models (out of 48 that were comparable) to fit the *D. pandora* transition data. All tested models used the Briere thermal performance curve. R-hat convergence is of the most poorly converged parameter in the model.

Competition function	Error Model	ΔELPD LOO	R-hat Convergence
BHTEMP1	Zero-inflated NB with competition-dependent shape	0	1.002
BHTEMP	Zero-inflated NB with competition-dependent shape	-0.53	1.002
BHTEMP1Beta	Zero-inflated NB with competition-dependent shape	-1.29	1.005
BHTEMPBeta	Zero-inflated NB with competition-dependent shape	-1.58	1.01
BH	Zero-inflated NB with competition-dependent shape	-2.15	1.002
BHBeta	Zero-inflated NB with competition-dependent shape	-2.72	1.002
BHTEMP1LogBeta	Zero-inflated NB with competition-dependent shape	-12.8	1.002
BHTEMP1	Zero-inflated NB with temperature-dependent shape	-26.1	1.001
BHTEMP	Zero-inflated NB with temperature-dependent shape	-26.3	1.001
BHTEMPBeta	Zero-inflated NB with temperature-dependent shape	-27.7	1.003
BHTEMP1Beta	Zero-inflated NB with temperature-dependent shape	-27.9	1.003
BH	Zero-inflated NB with temperature-dependent shape	-31.3	1.002
BHBeta	Zero-inflated NB with temperature-dependent shape	-31.8	1.002
BHTEMP1LogBeta	Zero-inflated NB with temperature-dependent shape	-34.5	1.001
BHTEMP1Log	Zero-inflated NB with competition-dependent shape	-39.6	1.001
BH	Temperature-dependent-zero-inflated NB	-50.9	1.002
BHTEMP1	Temperature-dependent-zero-inflated NB	-50.9	1.002
BHTEMP	Temperature-dependent-zero-inflated NB	-51.4	1.003
BHBeta	Temperature-dependent-zero-inflated NB	-51.4	1.003
BHTEMP1Beta	Temperature-dependent-zero-inflated NB	-51.9	1.002
BHTEMPBeta	Temperature-dependent-zero-inflated NB	-52	1.002
BHTEMP1LogBeta	Temperature-dependent-zero-inflated NB	-59.6	1.001
BHTEMP1Log	Zero-inflated NB with temperature-dependent shape	-64	1.001
BHTEMP1	Zero-inflated NB	-67.1	1.001
BHTEMP	Zero-inflated NB	-67.9	1.002

Table S5 Posterior summary of raw parameters within selected model for *D. pallidifrons*. Model followed: PAL_FEMALE ~ Prev_PAL_FEMALE * (B0 * PrevTemp * (PrevTemp - Tmin) * sqrt(Tmax - PrevTemp)) * (1/((1 + (alphaii0 + alphaiiT * Temp20) * Prev_PAL_FEMALE_comp + alphaij * Prev_PAN_FEMALE))) , zi ~ PrevTemp See code supplement for full model code and full posterior distributions. Note that to aid readability and make it easier to define weak priors on the intercept, the temperature value used to determine the effect of temperature on competition impacts was reduced by 20 to create the 'Temp20' variable.

Parameter	Estimate	Est.Error	l-95% CI	u-95% CI
B0	0.0795	0.0257	0.0332	0.13
Tmin	18.6	2.18	12.4	21
Tmax	28	0.0605	27.9	28.1
alphaii0	0.456	0.226	0.0555	0.921
alphaij	0.273	0.0954	0.106	0.478
alphaiiT	0.0955	0.0421	0.015	0.175
Zi_Intercept	-17.4	2.02	-21.4	-13.6
zi_PrevTemp	0.628	0.0788	0.48	0.784

Table S6 Posterior summary of raw parameters within selected model for *D. pandora*. Where model followed: PAN_FEMALE ~ Prev_PAN_FEMALE * (B0 * PrevTemp * (PrevTemp - Tmin) * sqrt(Tmax - PrevTemp)) * (1/((1 + alphaii * Prev_PAN_FEMALE_comp + (alphaij0 + alphaijT * Temp20) * Prev_PAL_FEMALE))) , shape ~ Prev_PAN_FEMALE See code supplement for full model code and full posterior distributions. Note that to aid readability and make it easier to define weak priors on the intercept, the temperature value used to determine the effect of temperature on competition impacts was reduced by 20 to create the 'Temp20' variable.

Parameter	Estimate	Est.Error	l-95% CI	u-95% CI
B0	0.0149	0.00929	0.0043	0.0393
Tmin	17.8	2.73	11.2	21.5
Tmax	33.6	2.99	29.5	39.5
alphaii	0.173	0.0566	0.0974	0.314
alphaij0	0.736	0.22	0.341	1.21
alphaijT	-0.0607	0.0336	-0.123	0.00964
Shape_intercept	-1.14	0.114	-1.36	-0.903
shape_Prev_PAN_FEMALE	0.0505	0.00403	0.0424	0.0583

Model	Competition from PAN	Environment	Constituent Partitions
A (Baseline)	None	Fixed	r_0
B	Constant (carrying capacity at constant environment)	Fixed	$r_0 + \Delta_C$
C	None	Fluctuating	$r_0 + \Delta_\sigma$
D	Present (variable following fluctuations)	Fluctuating (uncorrelated between focal and competitor)	$r_0 + \Delta_C + \Delta_\sigma + \Delta_*$
E (Full)	Present (variable following fluctuations)	Fluctuating (Same sequence affects both focal and competitor)	$r_0 + \Delta_C + \Delta_\sigma + \Delta_* + \Delta_{cov}$

Table S7 Models compared to partition contribution of processes to changes in the predicted long-term growth rate. Care must be taken when comparing between studies as different choices in how the environmental variation impacts the competitor species density can create additional small interactive effects.

SI Bibliography

1. Kleiber, C. & Zeileis, A. Visualizing Count Data Regressions Using Rootograms. *The American Statistician* **70**, 296–303 (2016).
